# Calmodulin Bidirectionally Regulates Evoked and Spontaneous Neurotransmitter Release at Retinal Ribbon Synapses

**DOI:** 10.1523/ENEURO.0257-20.2020

**Published:** 2021-01-05

**Authors:** Chao-Qun Liang, Gong Zhang, Lei Zhang, Si-Yun Chen, Jun-Nan Wang, Ting-Ting Zhang, Joshua H. Singer, Jiang-Bin Ke

**Affiliations:** 1 State Key Laboratory of Ophthalmology, Zhongshan Ophthalmic Center, Sun Yat-sen University, Guangzhou 510060, China; 2 Department of Biology, University of Maryland, College Park, MD 20742

**Keywords:** AII amacrine cell, calmodulin, myosin light chain kinase, neurotransmitter release, retinal rod bipolar cell, ribbon synapse

## Abstract

For decades, a role for the Ca^2+^-binding protein calmodulin (CaM) in Ca^2+^-dependent presynaptic modulation of synaptic transmission has been recognized. Here, we investigated the influence of CaM on evoked and spontaneous neurotransmission at rod bipolar (RB) cell→AII amacrine cell synapses in the mouse retina. Our work was motivated by the observations that expression of CaM in RB axon terminals is extremely high and that [Ca^2+^] in RB terminals normally rises sufficiently to saturate endogenous buffers, making tonic CaM activation likely. Taking advantage of a model in which RBs can be stimulated by expressed channelrhodopsin-2 (ChR2) to avoid dialysis of the presynaptic terminal, we found that inhibition of CaM dramatically decreased evoked release by inhibition of presynaptic Ca channels while at the same time potentiating both Ca^2+^-dependent and Ca^2+^-independent spontaneous release. Remarkably, inhibition of myosin light chain kinase (MLCK), but not other CaM-dependent targets, mimicked the effects of CaM inhibition on evoked and spontaneous release. Importantly, initial antagonism of CaM occluded the effect of subsequent inhibition of MLCK on spontaneous release. We conclude that CaM, by acting through MLCK, bidirectionally regulates evoked and spontaneous release at retinal ribbon synapses.

## Significance Statement

Calmodulin (CaM), a Ca^2+^-binding protein expressed throughout the CNS, functions as a presynaptic modulator of synaptic transmission. We showed that CaM was strongly expressed in the axon terminals of rod bipolar (RB) cells of the mouse retina. Inhibition of CaM decreased evoked release while potentiating spontaneous release at RB→AII amacrine cell synapses; this effect was mimicked by inhibition of myosin light chain kinase (MLCK), a CaM target. Thus, we conclude that CaM, acting through MLCK, bidirectionally regulates evoked and spontaneous release at retinal ribbon synapses.

## Introduction

At chemical synapses, fast, synchronous neurotransmitter release is evoked when membrane depolarization, usually in the form of action potentials, arrives at presynaptic axon terminals and opens voltage-gated calcium channels (VGCCs), permitting Ca^2+^ influx to trigger fusion of synaptic vesicles. Spontaneous neurotransmitter release also occurs independent from presynaptic activity, and understanding of its separate roles in synapse formation, synaptic maintenance and dendritic protein translation is emerging (Kavalali, 2015; Andreae and Burrone, 2018; Chanaday and Kavalali, 2018). Recent studies support the notion that there are distinct molecular mechanisms underlying evoked and spontaneous release: these two forms of neurotransmission may originate from separate synaptic vesicle pools (Chanaday and Kavalali, 2018), although evidence against this hypothesis also exists (Groemer and Klingauf, 2007; Hua et al., 2010; Wilhelm et al., 2010; Schneggenburger and Rosenmund, 2015).

Proteins such as Vti1a, VAMP7, Doc2 and copine-6 selectively regulate spontaneous release (Groffen et al., 2010; Pang et al., 2011; Ramirez et al., 2012; Bal et al., 2013; Liu et al., 2018), whereas others, e.g., RIM1 and RIM-binding proteins (RBPs), are essential for evoked but not spontaneous release (Calakos et al., 2004; Acuna et al., 2015; Robinson et al., 2019). Synaptotagmin-1 and complexins appear to promote evoked release but suppress spontaneous release (Maximov and Südhof, 2005; Huntwork and Littleton, 2007; Xu et al., 2009; Yang et al., 2013; Bai et al., 2016; Mortensen et al., 2016), although a more recent study argues against the role of complexins as “fusion clamps” for spontaneous release, and shows that knock-out of complexins reduces both spontaneous and evoked release (López-Murcia et al., 2019). Finally, neuromodulation can affect spontaneous and evoked release differentially: in L5 pyramidal cells of mouse brains, presynaptic NMDA receptors regulate evoked and spontaneous release via nonoverlapping RIM1-dependent and JNK2-dependent mechanisms, respectively (Abrahamsson et al., 2017).

Because they are designed to signal tonically over extended periods (Matthews and Fuchs, 2010; Cho and von Gersdorff, 2012), ribbon-type excitatory synapses experience sustained Ca^2+^ influx and elevations in intracellular [Ca^2+^] and appear to be a good model for understanding modulation of evoked and spontaneous release. Imaging of fish bipolar cells and salamander rod photoreceptors show that evoked release is concentrated near ribbon-type active zones (AZs) and spontaneous release occurs at a higher rate away from ribbon sites (Midorikawa et al., 2007; Zenisek, 2008; Chen et al., 2013), and electrophysiological analysis of transmission at mouse rod bipolar (RB)→AII synapses reveals two putatively separate pools of synaptic vesicles: ribbon-associated and ribbon independent (Mehta et al., 2014). Deletion of RIBEYE, a ribbon AZ-specific protein, results in an absence of ribbon AZs, as expected, and severely impairs evoked, but not spontaneous, release at RB→AII synapses (Maxeiner et al., 2016).

Here, we used the RB→AII synapse in the mouse retina, a well-established model ribbon-type synapse (Singer, 2007; Singer et al., 2009; Pangrsic et al., 2018), to examine a role for calmodulin (CaM) in the regulation of evoked and spontaneous transmission at synapses that experience tonic elevations in [Ca^2+^] and release neurotransmitter in a sustained manner over long periods.

CaM, a Ca^2+^-binding protein highly conserved among eukaryotes and expressed ubiquitously and abundantly in the brain, including the retina (Pochet et al., 1991), interacts with a large number of presynaptic targets, including, but not limited to, Ca^2+^/CaM-dependent kinase II (CaMKII), myosin light chain kinase (MLCK), calcineurin, Munc13, and Ca channels, all of which are involved in multiple mechanisms regulating the vesicle cycle (Hoeflich and Ikura, 2002; Ben-Johny and Yue, 2014; Chanaday and Kavalali, 2018; Tarasova et al., 2018). We used a variety of experimental approaches to demonstrate that CaM, by acting through MLCK, bidirectionally regulates evoked and spontaneous release at retinal ribbon-type synapses.

## Materials and Methods

### Animals

All animal procedures were performed in accordance with the University of Maryland and Sun Yat-sen University animal care committees’ regulations. Four transgenic mouse lines, two cre driver lines and two reporter lines, were used. RBs were targeted for transgene expression in the Pcp2-cre [Tg(Pcp2-cre)1Amc/J; Jax 006207] and BAC-Pcp2-IRES-Cre [B6.Cg-Tg(Pcp2-cre)3555Jdhu/J; Jax 010536] lines, both of which express cre recombinase primarily in RBs under the control of mouse Purkinje cell protein (Pcp2; Zhang et al., 2005; Ivanova et al., 2010). Channelrhodopsin-2 (ChR2) or GCaMP3 were expressed in RBs by cre-dependent recombination after crossing a cre driver line with either Ai32 [B6.Cg-Gt(ROSA)26Sor^tm32(CAG-COP4^*^H134R/EYFP)Hze^/J; Jax 012569] or Ai38 [B6;129S-Gt(ROSA)26Sor^tm38(CAG-GCaMP3)Hze^/J; Jax 014538] lines, respectively (Madisen et al., 2012; Zariwala et al., 2012). The wild-type (wt) C57BL/6J, Pcp2-cre::Ai32 and Pcp2-cre::Ai38 mice of either sex at ages between 4 and 20 weeks were used in this study.

### Electrophysiology

Retinal slices (200-μm thickness) were prepared from light-adapted retina isolated from either Pcp2-cre::Ai32 or wt mice. A retina was isolated into sodium bicarbonate buffered Ames’ medium (Sigma) equilibrated with 95% O_2_ and 5% CO_2_ (carbogen) at room temperature. For slice preparation, the retina was embedded in low-melting temperature agarose (Sigma Type VIIA, 2–3% in a HEPES-buffered saline), and slices were cut on a vibrating microtome (Leica VT1200s). Slices were stored in carbogen-bubbled Ames’ medium at room temperature until use.

Recordings were performed at near-physiological temperature (30–35°C). Retinal slices were continuously superfused at a rate of 1–2 ml/min with carbogen-bubbled artificial CSF (ACSF) containing the following: 119 mm NaCl, 23 mm NaHCO_3_, 10 mm glucose, 1.25 mm NaH_2_PO_4_, 2.5 mm KCl, 1.15 mm CaCl_2_, 1.5 mm MgCl_2_, 2 mm NaLactate, and 2 mm NaPyruvate. Picrotoxin (50 μm), (1,2,5,6-tetrahydropyridin-4-yl) methylphosphinic acid (TPMPA, 50 μm) or 4-imidazoleacetic acid (I4AA, 10 μm), strychnine (0.5 μm), tetrodotoxin (TTX; 0.5 μm), 2-amino-4-phosphonobutyate (L-AP4, 2 μm), and (*S*)−1-(2-amino-2-carboxyethyl)−3-(2-carboxy-5-phenylthiophene-3-yl-methyl)−5-methylpyrimidine-2,4-dione (ACET, 1 μm) were added to the ACSF to block GABA_A_R-mediated, GABA_C_R-mediated, GlyR-mediated, voltage-gated Na channel-mediated, mGluR6-regulated channel-mediated, and kainate receptor-mediated currents, respectively.

During current-clamp recordings, pipettes were filled with the following: 110 mm K-gluconate, 5 mm NaCl, 10 mm HEPES, 8 mm Tris-phosphocreatine, 4 mm Mg-ATP, 0.4 mm Na-GTP, and 1 mm BAPTA. The pH value was adjusted to 7.2 with KOH and osmolarity to ∼285 mOsm with sucrose. During voltage-clamp recordings, pipettes were filled with the following: 95 mm Cs-methanesulfonate, 20 mm TEA-Cl, 1 mm 4-AP, 10 mm HEPES, 8 mm Tris-phosphocreatine, 4  mm Mg-ATP, 0.4 mm Na-GTP, and 1 mm BAPTA. The pH value was adjusted to 7.2 with CsOH and osmolarity to ∼285 mOsm with sucrose. Alexa Fluor 594 or 647 was included in the pipette solutions to visualize the cell morphology after recordings. Generally, RB holding potential was −60 mV and AII holding potential was −80 mV, and membrane potentials were corrected for junction potentials of ∼−10 mV. Access resistances were <25 ΜΩ for RBs and < 20 ΜΩ for AII amacrine cells and were compensated by 50–90%. Recordings were made using MultiClamp 700B amplifiers. Recorded currents were digitized at 10–20 kHz and low-pass filtered at 2 kHz by an ITC-18 A/D board (Heka/Instrutech) controlled by software written in Igor Pro 6 (WaveMetrics). Recorded RB Ca currents were leak-subtracted off-line (P/4 protocol). Analysis was performed in Igor Pro.

### Optogenetics

ChR2 was activated by a high-power blue LED (Thor Laboratories; λ_peak_ 470 nm) directed through the 60× or 100× lenses to create a light spot (125 or 75 μm in diameter, respectively). The light intensities and durations were controlled by software written in Igor Pro.

### Calcium imaging

Retinal slices (200-μm thickness) were prepared, as described above, from light-adapted retina isolated from Pcp2-cre::Ai38 mice. Imaging of GCaMP3 signals was performed using two-photon laser-scanning microscopy (2PLSM). GCaMP3 was excited using a pulsed infrared laser (Chameleon; Coherent) tuned to a 910-nm excitation wavelength, and emitted light was passed through a series of dichroic mirrors and filters and collected by GaAsP photomultiplier tubes (Thor Laboratories). Frames containing multiple RB axon terminals in each of which several varicosities were evident were collected at ∼19 Hz and every nine frames were averaged to generate each calcium imaging picture shown below. Collected data were analyzed using ImageJ (Schneider et al., 2012).

### Immunohistochemistry

Retinas from wt mice were dissected and fixed in 4% PFA for 20 min. After fixation, retinas were infiltrated with graded (10%, 20%, and 30%) sucrose in PB and sectioned vertically at 14 μm on a cryostat (Leica). Immunohistochemical labeling was conducted by using the indirect fluorescence method. Retinal sections were blocked in 1% bovine serum albumin (BSA) in 0.1% Triton X-100 in PBS (PBST) for 2 h. Following removal of the blocking solutions, retinal sections were incubated with primary antibodies overnight at 4°C. The following primary antibodies were used: rabbit anti-CaM (1:500, #301003, Synaptic Systems), mouse anti-PKCα (1:200, #P5704, Sigma), and mouse anti-CtBP2 (1:500, #612004, BD Biosciences). After rinsing, corresponding secondary antibodies including Alexa Fluor 488 donkey anti-rabbit (1:200, #A21206, Thermo Fisher Scientific) and Alexa Fluor 568 donkey anti-mouse (1:200, #A10037, Thermo Fisher Scientific) were applied for 2 h in darkness. All antibodies were diluted with 5% BSA in PBST. After rinsing, DAPI stain (1:1000, #C1002, Beyotime) was applied for 10 min at room temperature. Between incubations, sections were washed three times for 5 min each using PBST. Control experiments were conducted either by preincubation of the anti-CaM antibody with the CaM or Ca^2+^-binding protein 5 (CaBP5) immunopeptides (EEFVQMMTAK corresponding to AA 140–149 in rat/mouse CaM, #301-0P, Synaptic Systems; EEFVKMMSR corresponding to AA 165–173 in mouse CaBP5, synthesized by Sangon Biotech) or by omission of both primary antibodies. All labeled sections were examined with a confocal laser scanning microscope (LSM 880, Carl Zeiss) with Plan-Apochromat 63×/1.4 or 100×/1.40 oil-immersion objectives. Images were adjusted for contrast and brightness by using ZEN software (Carl Zeiss) or Photoshop software (Adobe Systems).

### Chemicals

ACET, picrotoxin, TPMPA, strychnine, DNQX, W-7, calmidazolium (CMZ), CALP1, ML-9, KN-62, MMPX, ascomycin, BAPTA-AM, thapsigargin (Tg), YM-58483 were obtained from Tocris. L-AP4 was purchased from Tocris or Cayman. Meclofenamic acid (MFA) and I4AA were obtained from Sigma. TTX was purchased from Alomone Labs. Drugs were dissolved in dimethylsulfoxide (DMSO) where appropriate and then diluted into the bath solution. The final concentrations of DMSO were <0.1% (v/v) in all experiments.

### Statistical analysis

Statistical analysis was performed with Prism 6 (GraphPad software). For better comparison among different groups, data acquired in each cell were normalized to the value under control condition. The Kolmogorov–Smirnov (KS) test was used to compare the cumulative distributions of data. Differences between experimental samples were assessed for significance using two-tailed Student’s *t* test, Wilcoxon signed-rank test or Mann–Whitney test where appropriate. Significance was taken as *p* < 0.05. All data were illustrated as mean ± SEM.

## Results

### Optogenetic control of transmission at the RB→AII synapse

**Figure 1.**
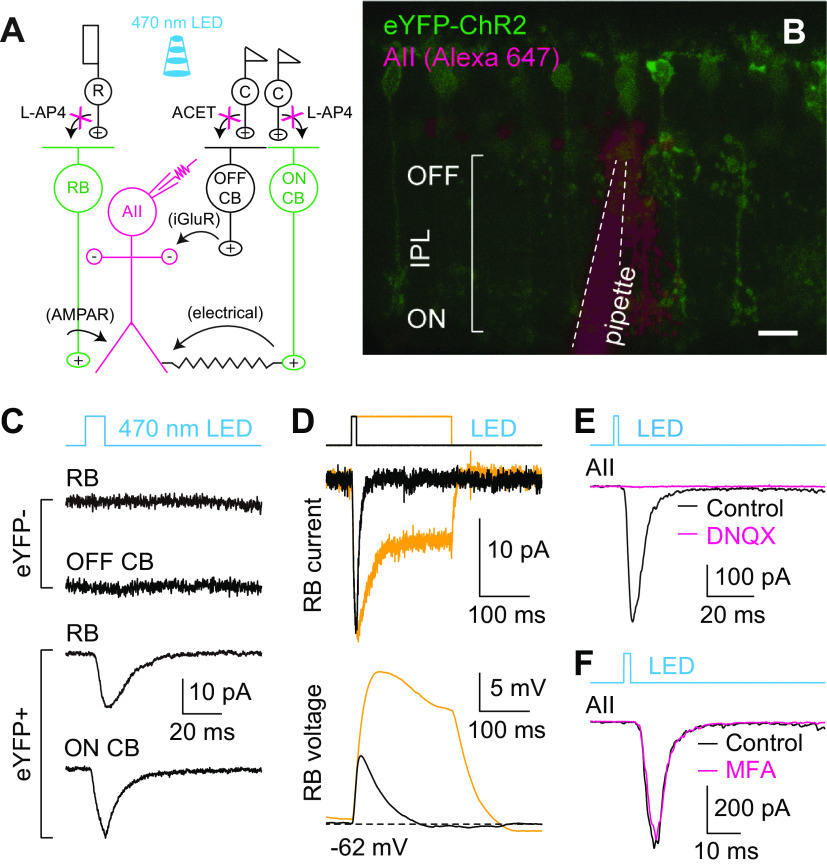
Optogenetic study of transmission at RB→AII synapses. ***A***, A diagram illustrating optogenetic study of synaptic transmission between RB cells and AII amacrine cells in the retinas of Pcp2-cre::Ai32 mice. After synaptic transmission between photoreceptors and bipolar cells is blocked with L-AP4 and ACET, 470-nm light flashes could stimulate light-sensitive ChR2 channels and thus directly activate ChR2-eYFP-expressing RBs and ON CB cells (green), and finally evoked responses in postsynaptic AII amacrine cells (magenta) could be recorded. ***B***, A two-photon image showing ChR2-eYFP expression (green) in a retinal slice made from a Pcp2-cre::Ai32 mouse; an AII amacrine cell (magenta) was recorded and filled with Alexa Fluor 647 by a patch pipette (outlined by dashed lines). Scale bar: 10 μm. ***C***, Representative traces showing ChR2-mediated currents recorded in an eYFP+ RB and an eYFP+ ON CB, but not in either eYFP– RB or eYFP– OFF CB, during brief flashes of 470-nm LED. V_hold_ = −60 mV. ***D***, Representative traces showing ChR2-mediated current (voltage-clamp mode; V_hold_ = −60 mV) and membrane potential (voltage; current-clamp mode) changes recorded in an eYFP+ RB during brief (10 ms) and long (200 ms) light stimuli. ***E***, During brief flashes, the eEPSCs recorded in AII amacrine cells postsynaptic to RBs were blocked almost completely by DNQX (20 μm). ***F***, The eEPSCs recorded during brief flashes were only slightly influenced by the gap junction blocker, MFA (100 μm).

To permit transmission at RB→AII synapses to be evoked reliably over extended experimental periods, we used an optogenetic approach in which ChR2 was expressed in RBs by cre-mediated recombination in Pcp2-cre::Ai32 mouse retinas (Fig. 1*A*). As reported previously (Zhang et al., 2005; Ivanova et al., 2010), expression of ChR2-eYFP was observed in ON bipolar cells, largely RBs, in *in vitro* retinal slices prepared from these animals (Fig. 1*B*). With synaptic transmission between photoreceptors and bipolar cells blocked with the mGluR6 agonist L-AP4 (2 μm; to block photoreceptor→ON bipolar cell synapses, including rod→RB synapses) and the kainate receptor antagonist ACET [1 μm; to block photoreceptor→OFF cone bipolar (CB) cell synapses; Park et al., 2018, 2020], 470-nm light induced ChR2-mediated currents in eYFP+ RBs and ON CBs, but not in eYFP– RBs and OFF CBs (Fig. 1*C*). Changes in light intensity and stimulus duration modulated ChR2-mediated current and membrane potential changes in RBs (Fig. 1*D*). Most importantly, brief flashes (e.g., 1–10 ms) evoked EPSCs in AII amacrine cells. The EPSCs recorded in AIIs were abolished almost completely by non-NMDA glutamate receptor antagonist DNQX (20 μm; Fig. 1*E*), which blocks transmission at RB→AII synapses (Singer and Diamond, 2003). In contrast, the EPSCs were only slightly affected by MFA (100 μm; Fig. 1*F*), which blocks the gap junctions between AII amacrine cells and ON CBs (Veruki and Hartveit, 2009). Overall, these results indicated that optogenetic control of transmission at RB→AII synapse was achieved reliably. Light-evoked EPSCs (termed eEPSCs hereafter) were quite stable over long periods (>20 min), which allowed us to assess regulation of neurotransmitter release at RB→AII synapses.

### A CaM antagonist affects evoked and spontaneous release differently

Physiologic depolarization of RB terminals results in Ca^2+^ influx sufficient to raise intracellular [Ca^2+^] to levels that saturate endogenous buffers (Mehta et al., 2014). Given that CaM is both a well-characterized Ca^2+^ sensor and a modulator of synaptic transmission (Chanaday and Kavalali, 2018), we postulated that were CaM expressed in RB terminals, it would play a significant role in modulating transmission at RB→AII synapses.

**Figure 2.**
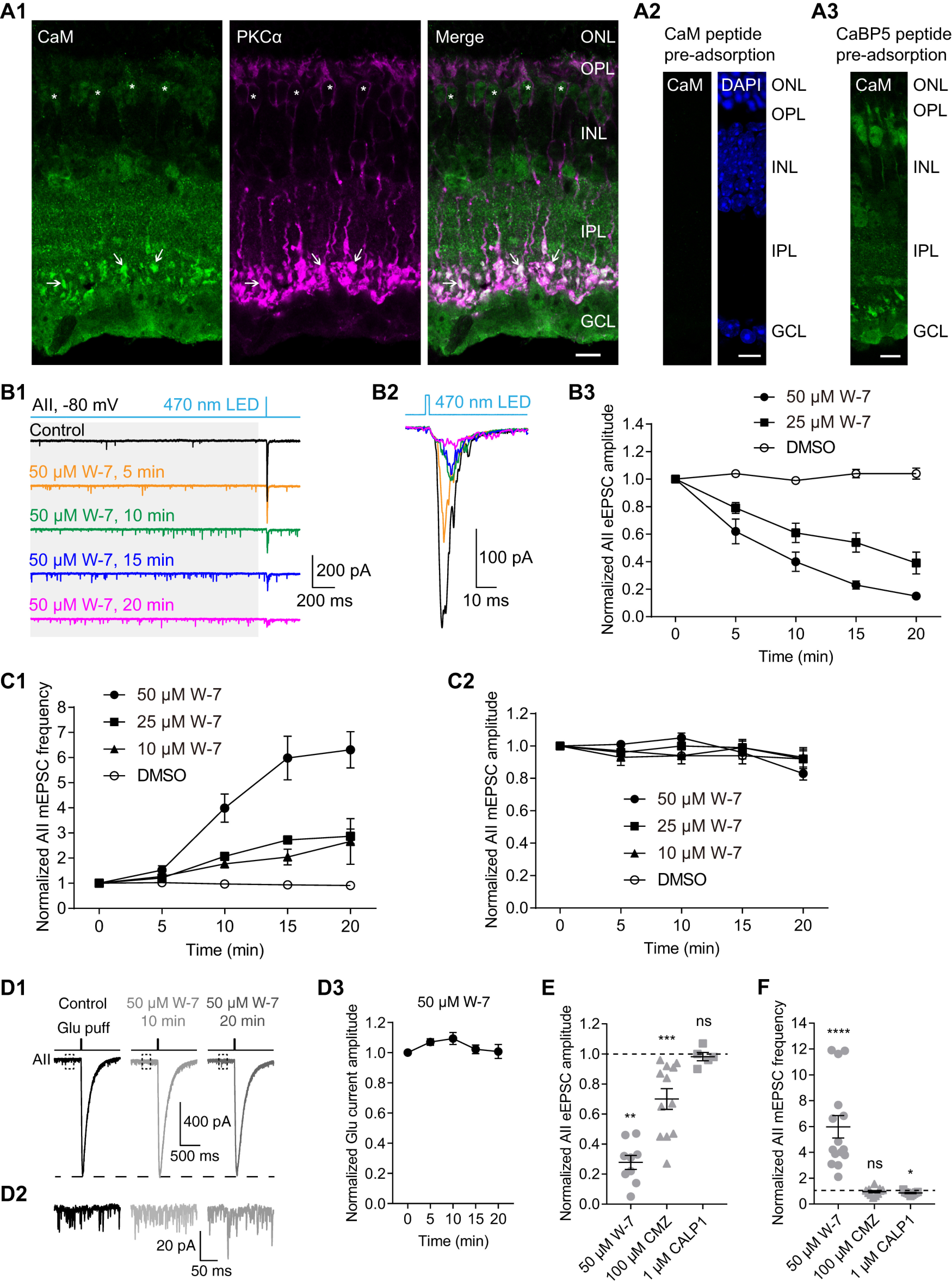
CaM bidirectionally regulates evoked and spontaneous neurotransmitter release from RBs. ***A1***, Confocal images showing immunofluorescence double labeling of CaM (green) and PKCα (magenta), a specific cell marker of RBs, in a frozen retinal slice. In the merged image (green + magenta), expression of CaM could be clearly seen in the axon terminals (arrow) and somata (asterisk) of RBs. Scale bar: 10 μm. ONL: outer nuclear layer; OPL: outer plexiform layer; INL: inner nuclear layer; IPL: inner plexiform layer; GCL: ganglion cell layer. ***A2***, No labeling was observed in the negative control where the anti-CaM antibody was preincubated with the CaM immunopeptide. DAPI staining showed the three major cell body layers in the retina. Scale bar: 10 μm. ***A3***, Preadsorption of the anti-CaM antibody with the CaBP5 immunopeptide did not change the staining pattern for CaM. Scale bar: 10 μm. ***B1***, Two-millisecond flashes of 470-nm LED were presented to stimulate ChR2 in Pcp2-cre::Ai32 mice with L-AP4 and ACET in the bath to block synaptic transmission between photoreceptors and bipolar cells; all the inhibitory connections were also blocked. The evoked responses (eEPSCs) and the small responses induced by spontaneous release before light onset (mEPSCs; see gray background area) in AII amacrine cells were recorded. V_hold_ = −80 mV. Individual traces showed that the CaM antagonist, W-7 (50 μm) strongly increased mEPSC frequency and reduced eEPSC amplitude. ***B2***, Average traces of eEPSCs recorded in the same AII in ***B1***. ***B3***, Statistics of the effects of 25 μm (*n* = 6) and 50 μm (*n* = 9) W-7 on eEPSC amplitude. The amplitudes were normalized to the amplitude at time 0 in each cell before averaging across cells. ***C1***, Statistics of the effects of 10 μm (*n* = 8), 25 μm (*n* = 13), and 50 μm (*n* = 15) W-7 on mEPSC frequency. The frequencies were normalized to the frequency at time 0 in each cell before averaging across cells. ***C2***, Statistics of the effects of 10 μm (*n* = 8), 25 μm (*n* = 13), and 50 μm (*n* = 15) W-7 on mEPSC amplitude. The amplitudes were normalized to the amplitude at time 0 in each cell before averaging across cells. ***D1***, Individual traces showing that W-7 (50 μm) had no inhibitory effect on AMPA receptor-mediated currents recorded in an AII evoked by glutamate (1 mm) applied onto the AII dendrites at the border of the IPL and GCL. V_hold_ = −80 mV. ***D2***, Magnification of the traces in the dashed line frames of ***D1***, showing increase of mEPSC frequency by W-7. ***D3***, Statistics of the effects of 50 μm W-7 (*n* = 7) on the amplitude of glutamate-evoked currents. The amplitudes were normalized to the amplitude at time 0 in each cell before averaging across cells. ***E***, Summary data showing the effects of 50 μm W-7 (circles), 100 μm CMZ (triangles), another CaM antagonist, and 1 μm CALP1 (squares), a CaM agonist, on eEPSC amplitude after bath application for 15 min. The amplitudes were normalized to the amplitude at time 0 in each cell before averaging across cells. The data were also illustrated as mean ± SEM. Wilcoxon signed-rank tests were used (control vs W-7, *n* = 9, *p* = 0.0039; control vs CMZ, *n* = 12, *p* = 0.0005; control vs CALP1, *n* = 5, *p* = 0.6250); **p < 0.01, ***p < 0.001; ns: not statistically different. Note that CMZ reduced eEPSC amplitude too, but CALP1 did not enhance eEPSC amplitude under control conditions. ***F***, Summary data showing the effects of 50 μm W-7 (circles), 100 μm CMZ (triangles), and 1 μm CALP1 (squares) on mEPSC frequency. The frequencies were normalized to the frequency at time 0 in each cell before averaging across cells. The data were also illustrated as mean ± SEM. Wilcoxon signed-rank tests were used (control vs W-7, *n* = 15, *p* < 0.0001; control vs CMZ, *n* = 12, *p* = 0.6377; control vs CALP1, *n* = 9, *p* = 0.0273); *p < 0.05, ****p < 0.0001; ns: not statistically different. Note that CMZ did not change the mEPSC frequency, but activation of CaM by CALP1 slightly reduced mEPSC frequency.

We therefore performed immunofluorescence double labeling of CaM and PKCα (a specific marker of RBs), and we found that CaM was strongly expressed in the axon terminals and, to a lesser extent, in the somata of RBs (Fig. 2*A1*,*A2*). Interestingly, RBs seemed to have the highest CaM expression level, especially in their axon terminals, among all retinal cell types, suggesting that CaM might play a prominent role in presynaptic functions (Pangrsic et al., 2018).

It has been reported that CaBP5, with ∼58% sequence similarity to CaM, also is expressed in mouse RBs; because it interacts with Munc18-1 and myosin VI, both of which are involved in synaptic vesicle cycle, it has been suggested that CaBP5 may play a modulatory role in synaptic transmission (Haeseleer et al., 2000; Rieke et al., 2008; Sokal and Haeseleer, 2011). Preincubation of the anti-CaM antibody with the CaBP5 peptide, however, did not change the staining pattern for CaM as the CaM peptide did (Fig. 2*A2*,*A3*), indicating that the anti-CaM antibody used in this study did not cross-react with CaBP5 proteins.

We recorded miniature EPSCs (mEPSCs) and eEPSCs in AIIs, which reflect spontaneous and evoked neurotransmitter release from RBs, respectively, and assessed the effect of modulating CaM. Remarkably, bath application of a CaM antagonist, W-7 (50 μm) had strong, distinct effects on mEPSCs and eEPSCs: W-7 strongly increased mEPSC frequency (Fig. 2*B1*) while dramatically suppressing eEPSC amplitude (Fig. 2*B1*,*B2*). We also tested the effect of W-7 on mEPSCs in wt mice. Since there were no visible differences between the Pcp2-cre::Ai32 and wt mouse data, they were pooled. The effects of W-7 on eEPSCs (*n* = 9 for 50 μm; *n* = 6 for 25 μm; Fig. 2*B3*) and mEPSCs (*n* = 15 for 50 μm; *n* = 13 for 25 μm; *n* = 8 for 10 μm; Fig. 2*C1*) were both time and concentration dependent. W-7, however, had no obvious effect on mEPSC amplitude (*n* = 15 for 50 μm; *n* = 13 for 25 μm; *n* = 8 for 10 μm; Fig. 2*C2*), indicating that inhibition of presynaptic CaM strongly enhanced spontaneous release from RB terminals. Note that DMSO alone, at the same concentration as was present with 50 μm W-7, did not change the eEPSC amplitude (Fig. 2*B3*), mEPSC frequency (Fig. 2*C1*), or mEPSC amplitude (Fig. 2*C2*).

To strengthen our conclusion that suppression of eEPSCs by W-7 arose from a presynaptic mechanism, we recorded the postsynaptic AMPA receptor-mediated currents in AIIs evoked by glutamate (1 mm) applied directly onto the AII dendrites located at the border of the IPL and ganglion cell layer (GCL) when CoCl_2_ (1 mm) was included in the external solution to block all Ca^2+^-dependent synaptic transmission: 50 μm W-7 had no significant effect on the glutamate-evoked postsynaptic currents (*n* = 7; Figs. 2*D1*,*D3*). Notably, although CoCl_2_ should have blocked all VGCCs, W-7 still increased mEPSC frequency (Fig. 2*D2*); this result is explored further, below.

We also tested the effects of CMZ, another CaM antagonist, and CALP1, a CaM agonist (Villain et al., 2000), on eEPSCs and mEPSCs. Application of CMZ (100 μm), to a lesser extent, reduced eEPSC amplitude (control vs CMZ, 1.00 vs 0.70, *n* = 12 and *p* = 0.0005 by Wilcoxon signed-rank test; control vs W-7, 1.00 vs 0.28, *n* = 9 and *p* = 0.0039 by Wilcoxon signed-rank test; Fig. 2*E*), but it did not change the mEPSC frequency (control vs CMZ, 1.00 vs 0.96, *n* = 11 and *p* = 0.6377 by Wilcoxon signed-rank test; Fig. 2*F*). Application of CALP1 (1 μm) for 15 min, however, did not change the eEPSC amplitude (control vs CALP1, 1.00 vs 0.98, *n* = 5 and *p* = 0.6250 by Wilcoxon signed-rank test; Fig. 2*E*), but it slightly reduced mEPSC frequency (control vs CALP1, 1.00 vs 0.85, *n* = 9 and *p* = 0.0273 by Wilcoxon signed-rank test; by contrast, control vs W-7, 1.00 vs 5.95, *n* = 15 and *p* < 0.0001 by Wilcoxon signed-rank test; Fig. 2*F*). These results suggest that CaM is activated tonically by physiological Ca^2+^ influx into RB terminals under our experimental conditions.

**Figure 3.**
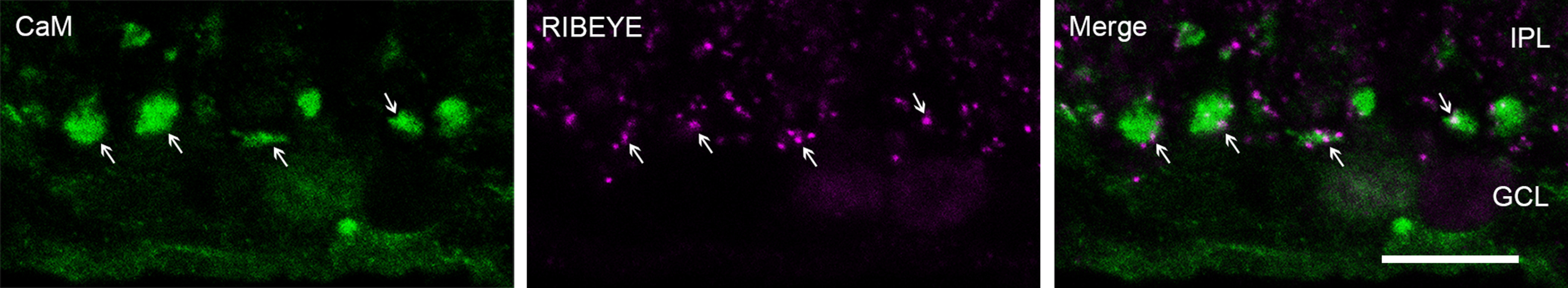
CaM is ubiquitously expressed at sites both near and away from ribbons in RB axon terminals. Confocal images showing immunofluorescence double labeling of CaM (green) and RIBEYE (magenta), a ribbon-specific protein, in the axon terminals of RBs. In the merged image (green + magenta), expression of CaM can be seen near ribbons (arrow) and away from ribbon sites. IPL: inner plexiform layer; GCL: ganglion cell layer. Scale bar: 10 μm.

Inhibition of CaM inhibits evoked transmission and stimulates spontaneous release at RB→AII synapses. Imaging exocytosis from bipolar cell terminals has led to the conclusion that evoked and spontaneous release might arise from physically separate presynaptic sites (Midorikawa et al., 2007; Zenisek, 2008), with spontaneous release occurring farther from the ribbon-type AZ than evoked release. The expression pattern of CaM in the axon terminals of RBs (Fig. 2*A1*) supports a role as a dual regulator of evoked and spontaneous release. Immunofluorescence double labeling of CaM and CtBP2/RIBEYE, a unique scaffolding protein of ribbons (Maxeiner et al., 2016), showed CaM to be expressed ubiquitously, at sites both near and away from ribbons (Fig. 3).

### Inhibition of CaM suppresses Ca currents in RB terminals

**Figure 4.**
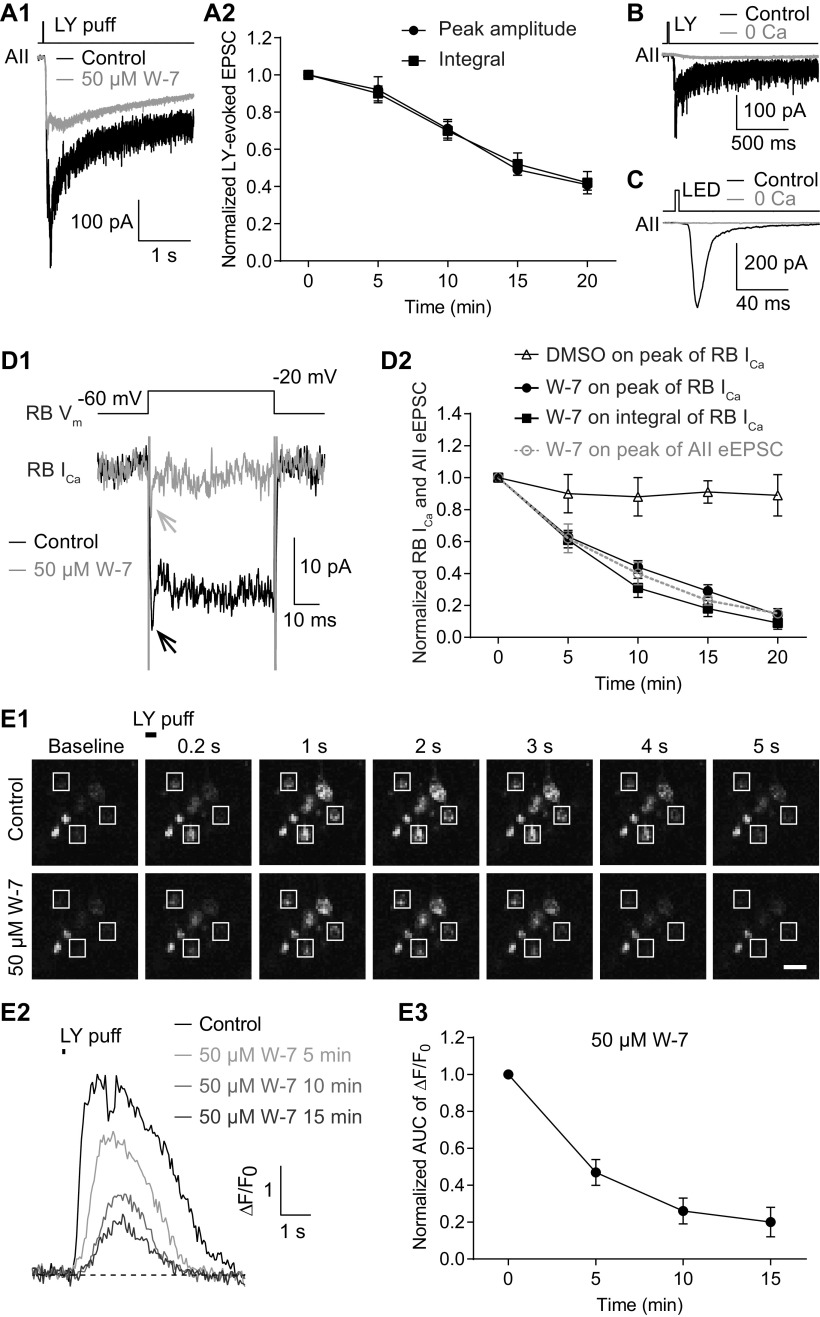
Inhibition of CaM strongly reduces evoked release from RBs by suppressing Ca^2+^ influx into their axon terminals. ***A1***, Average traces showing that EPSCs recorded in an AII, evoked by puffing LY 341495 (LY), an mGluR6 antagonist, onto the dendrites of RBs located at the OPL, were strongly reduced by application of 50 μm W-7. V_hold_ = −80 mV. ***A2***, LY-evoked EPSCs decreased over time with application of 50 μm W-7 (*n* = 7). The peak amplitudes and integrals of EPSCs were normalized to the amplitude and integral at time 0, respectively, in each cell before averaging across cells. ***B***, LY-evoked EPSCs were completely abolished by removing extracellular Ca^2+^ (0 Ca). ***C***, The EPSCs recorded in an AII, evoked by brief flashes of 470-nm LED in a Pcp2-cre::Ai32 mouse, were abolished completely by removing extracellular Ca^2+^ (0 Ca). ***D1***, Average traces showing that W-7 (50 μm) strongly suppressed the voltage step-generated Ca currents (I_Ca_) in an RB. ***D2***, Statistics of the effects of 50 μm W-7 (*n* = 7) and DMSO control on the peak amplitude and integral of RB I_Ca_. The suppression of I_Ca_ recorded in RBs was closely related to the inhibition of eEPSCs recorded in AIIs (data adapted from Fig. 2*B3*, superimposed in gray). ***E1***, Calcium imaging pictures from a Pcp2-cre::Ai38 mouse showing that LY-evoked changes of Ca^2+^ signals in RB axon terminals (white frames), detected by the Ca^2+^ indicator GCaMP3, were reduced strongly by application of 50 μm W-7. Scale bar: 1 μm. ***E2***, Representative ΔF/F_0_ traces showing suppression of Ca^2+^ signals in an RB axon terminal by 50 μm W-7. ***E3***, Summary data showing that LY-evoked Ca^2+^ signals in RB axon terminals (*n* = 21 terminals from 3 retinal slices), measured as areas under the curve (AUCs) of ΔF/F_0_ traces, were strongly suppressed by 50 μm W-7 over time. All the data were illustrated as mean ± SEM.

The similarity of the observations made in Pcp2-cre::Ai32 and wt retinas indicated that the effect of W-7 on spontaneous release was not influenced by exogenous expression of ChR2-eYFP in RBs. To further exclude the possibility that the effect of W-7 on evoked release might be because of exogenous ChR2-eYFP expression, we evoked EPSCs in AII amacrine cells from wt mice by puffing the mGluR6 antagonist LY 341495 (LY; 5–10 μm) onto RB dendrites located at the OPL, and then assessed the effect of applied W-7. Not surprisingly, W-7 (50 μm) strongly reduced the LY-evoked EPSCs (Fig. 4*A1*) with a seemingly slower time course (Fig. 4*A2*). We reasoned that LY application elicited larger changes in presynaptic [Ca^2+^] than did brief light stimuli, as evidenced from the large difference in the integrals of the EPSCs evoked by the two (integral of LY-evoked EPSCs, 830 737 ± 167 550, *n* = 7; integral of ChR2-driven EPSCs, 3929 ± 419, *n* = 9).

Given that both LY-evoked EPSCs (Fig. 4*B*) and light-evoked EPSCs (Fig. 4*C*) were abolished, as expected, by removal of extracellular Ca^2+^ (0 Ca^2+^; with 2 or 5 mm EGTA in the external solution), we tested the hypothesis that inhibition of CaM reduced evoked release by suppressing presynaptic Ca^2+^ influx. Depolarization of an RB with brief voltage step from −60 to −20 mV for 50 ms elicited sustained, inward Ca current (I_Ca_; 22.18 ± 2.16 pA, *n* = 7; Fig. 4*D1*). Both the peak amplitude and integral of RB I_Ca_ were reduced substantially by bath application of 50 μm W-7 (Fig. 4*D2*). The time course of suppression of RB I_Ca_ was strikingly consistent with that of inhibition of eEPSCs by 50 μm W-7 (Fig. 4*D2*). Application of DMSO alone seemed to reduce slightly but insignificantly the peak amplitude of RB I_Ca_ (Fig. 4*D2*).

To ensure that whole-cell recording from the RB did not alter modulation of presynaptic Ca channels, we imaged [Ca^2+^] in RB terminals of Pcp2-cre::Ai38 mice in which the fluorescent [Ca^2+^] indicator GCaMP3 was expressed (see Materials and Methods). Brief application (6–50 ms) of LY (5–10 μm) at the OPL depolarized RB dendrites, ultimately evoking strong fluorescence signals in RB axon terminals; these returned to baseline level after ∼5 s (Fig. 4*E1*, control). The LY-evoked Ca^2+^ signals, detected by GCaMP3, were strongly reduced by application of 50 μm W-7 (Fig. 4*E1–E3*), consistent with our observations of Ca currents (Fig. 4*D1*,*D2*).

Taken together, these results proved our hypothesis that inhibition of CaM strongly reduced evoked release from RBs by suppressing Ca^2+^ influx into axon terminals.

### Both Ca^2+^-dependent and Ca^2+^-independent spontaneous release are enhanced, with the latter to a greater extent, when CaM is inhibited

The presynaptic mechanisms underlying spontaneous release of neurotransmitters have been studied for decades (Fatt and Katz, 1950, 1952; Kavalali, 2015). Spontaneous release at RB→AII synapses depends on extracellular Ca^2+^ concentration, indicating that it is driven in part by Ca^2+^ influx through Ca channels that open spontaneously at resting potential (Singer et al., 2004; Mehta et al., 2013; Maxeiner et al., 2016; Mortensen et al., 2016). But, because spontaneous release from RB terminals persists at an appreciable rate when external Ca^2+^ is removed (Mortensen et al., 2016), there appears to be a Ca^2+^-independent component to the process.

**Figure 5.**
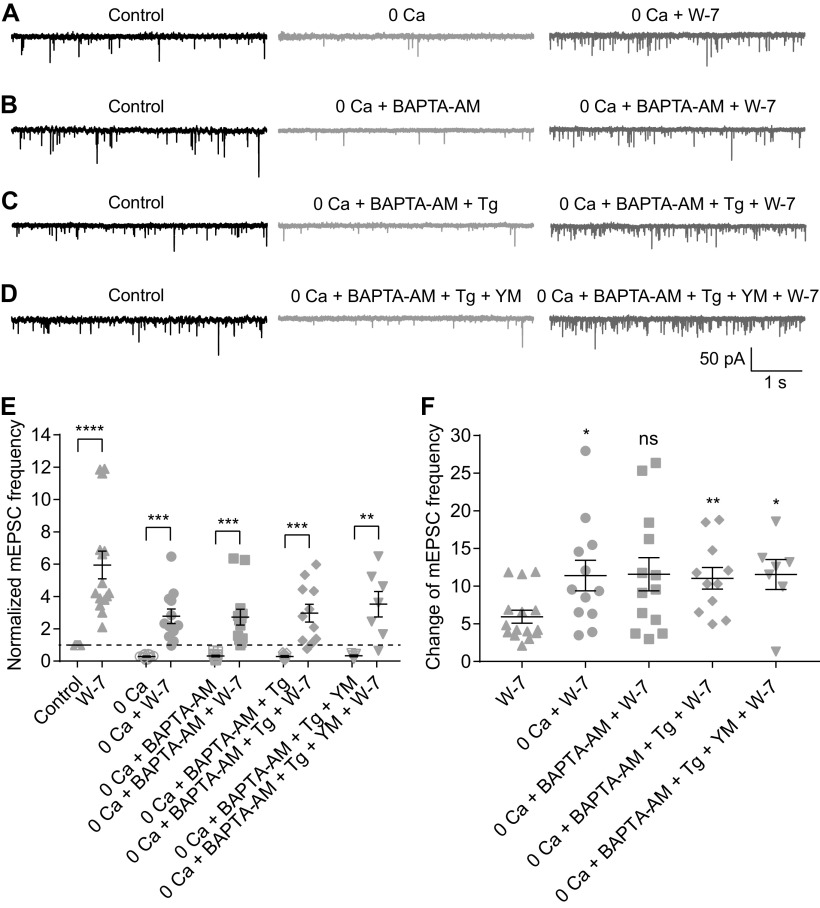
Both Ca^2+^-dependent and Ca^2+^-independent spontaneous release are enhanced, but to different extents, when CaM is inhibited. ***A–D***, Representative traces showing mEPSCs recorded in AIIs under four experimental conditions in different combinations of removal of extracellular calcium (0 Ca), 10 μm BAPTA-AM, 1 μm Tg, and 1 μm YM-58483 (YM). The traces showing mEPSCs after bath application of 50 μm W-7 for 15 min under each condition were also presented. ***E***, Summary data for AII mEPSC frequency under four experimental conditions in ***A–D***. The data under control and W-7 conditions (adapted from Fig. 2*C1*, empty and full up triangles, respectively) were also presented for direct comparison. The frequencies were normalized to the frequency under control condition in each cell before averaging across cells. The data were also illustrated as mean ± SEM. Paired Student’s *t* tests were used (0 Ca vs 0 Ca + W-7, *n* = 12, *p* = 0.0001; 0 Ca + BAPTA-AM vs 0 Ca + BAPTA-AM + W-7, *n* = 13, *p* = 0.0002; 0 Ca + BAPTA-AM + Tg vs 0 Ca + BAPTA-AM + Tg + W-7, *n* = 11, *p* = 0.0004; 0 Ca + BAPTA-AM + Tg + YM vs 0 Ca + BAPTA-AM + Tg + YM + W-7, *n* = 7, *p* = 0.0062) except for comparison of control and W-7 data by Wilcoxon signed-rank test (*n* = 15, *p* < 0.0001); **p < 0.01, ***p < 0.001, ****p < 0.0001. ***F***, Summary data for the relative effect of W-7 on AII mEPSC frequency under control and four experimental conditions. The change in each cell was calculated as the ratio of mEPSC frequencies before and 15 min after application of W-7. The data were also illustrated as mean ± SEM. Mann–Whitney tests were used (W-7 vs 0 Ca + W-7, *p* = 0.0214; W-7 vs 0 Ca + BAPTA-AM + W-7, *p* = 0.0954; W-7 vs 0 Ca + BAPTA-AM + Tg + W-7, *p* = 0.0045; W-7 vs 0 Ca + BAPTA-AM + Tg + YM + W-7, *p* = 0.0262); *p < 0.05, **p < 0.01; ns: not statistically different.

Given that W-7 (50 μm) increased mEPSC frequency after evoked transmission was blocked with CoCl_2_ (1 mm; Fig. 2*D2*), it appeared that inhibition of CaM enhanced Ca^2+^-independent spontaneous release. To validate this observation and to explore the potential underlying mechanisms, we tested the effects of 50 μm W-7 on mEPSCs under four experimental conditions: (1) removal of extracellular Ca^2+^ (0 Ca^2+^ plus 2 or 5 mm EGTA in the external solution; Fig. 5*A*); (2) 0 Ca^2+^ combined with bath application of 10 μm BAPTA-AM, a cell membrane permeable Ca^2+^ chelator with rapid binding kinetics (Fig. 5*B*); (3) 0 Ca^2+^, 10 μm BAPTA-AM, and 1 μm Tg, a sarco-endoplasmic reticulum Ca^2+^-ATPase inhibitor which depletes intracellular Ca^2+^ stores (Fig. 5*C*); and (4) 0 Ca^2+^, 10 μm BAPTA-AM, 1 μm Tg, and 1 μm YM-58483, which inhibits calcium release-activated calcium channels (CRACs) and suppresses Tg-induced Ca^2+^ influx (Fig. 5*D*). For each condition, mEPSCs were recorded for at least 15 min before bath application of 50 μm W-7. We analyzed mEPSCs recorded under control, experimental (one of the four detailed above), and experimental + W-7 conditions. Under each of the four experimental conditions, mEPSC frequency was reduced strongly compared with control, but for each of the four, application of 50 μm W-7 increased mEPSC frequency substantially (Fig. 5*E*; Table 1). The ratio of mEPSC frequencies before and after application of W-7 under each condition was also calculated (Fig. 5*F*; Table 1).

**Table 1:** The effects of W-7 on AII mEPSC frequency under different conditions

	Data	Data structure	Type of test	Power	Mean ± SEM	Number of cells
	Control	Non-normal distribution			1.00 ± 0.00	15
	50 μm W-7	Non-normal distribution			5.95 ± 0.86	15
a	Control vs W-7		Wilcoxon signed-rank test	*p* < 0.0001		
	0 Ca^2+^ (experimental condition 1)	Normal distribution			0.28 ± 0.03	12
	0 Ca^2+^ + W-7	Normal distribution			2.78 ± 0.45	12
b	0 Ca^2+^ vs 0 Ca^2+^ + W-7		Paired *t* test	*p* = 0.0001		
	0 Ca^2+^ + BAPTA-AM (experimental condition 2)	Normal distribution			0.32 ± 0.05	13
	0 Ca^2+^ + BAPTA-AM + W-7	Normal distribution			2.73 ± 0.49	13
c	0 Ca^2+^ + BAPTA-AM vs 0 Ca^2+^ + BAPTA-AM + W-7		Paired *t* test	*p* = 0.0002		
	0 Ca^2+^ + BAPTA-AM + Tg (experimental condition 3)	Normal distribution			0.29 ± 0.05	11
	0 Ca^2+^ + BAPTA-AM + Tg + W-7	Normal distribution			2.97 ± 0.55	11
d	0 Ca^2+^ + BAPTA-AM + Tg vs 0 Ca^2+^ + BAPTA-AM + Tg + W-7		Paired *t* test	*p* = 0.0004		
	0 Ca^2+^ + BAPTA-AM + Tg + YM (experimental condition 4)	Normal distribution			0.33 ± 0.05	7
	0 Ca^2+^ + BAPTA-AM + Tg + YM + W-7	Normal distribution			3.52 ± 0.78	7
e	0 Ca^2+^ + BAPTA-AM + Tg + YM vs 0 Ca^2+^ + BAPTA-AM + Tg + YM + W-7		Paired *t* test	*p* = 0.0062		
	Relative effect of W-7 under different conditions					
	W-7 effect under control condition	Non-normal distribution			5.95 ± 0.86	15
	W-7 effect under experimental condition 1	Normal distribution			11.41 ± 2.03	12
	W-7 effect under experimental condition 2	Normal distribution			11.59 ± 2.19	13
	W-7 effect under experimental condition 3	Normal distribution			11.05 ± 1.45	11
	W-7 effect under experimental condition 4	Normal distribution			11.56 ± 1.98	7
f	W-7 (experimental condition 1) vs W-7 (control condition)		Mann–Whitney test	*p* = 0.0214		
g	W-7 (experimental condition 2) vs W-7 (control condition)		Mann–Whitney test	*p* = 0.0954		
h	W-7 (experimental condition 3) vs W-7 (control condition)		Mann–Whitney test	*p* = 0.0045		
i	W-7 (experimental condition 4) vs W-7 (control condition)		Mann–Whitney test	*p* = 0.0262		

0 Ca^2+^: remove extracellular Ca^2+^; YM: YM-58483.

Additionally, we found that under experimental condition 1 (i.e., 0 Ca^2+^ condition), 1 μm CALP1 reduced average mEPSC frequency to ∼53% of control (*n* = 8; *p* = 0.0078 by Wilcoxon signed-rank test; control condition vs experimental condition 1, *p* = 0.0027 by unpaired Student’s *t* test). Interestingly, the relative effect on mEPSC frequency of either W-7 or CALP1 was stronger under experimental than control conditions, indicating that modulation of CaM influenced Ca^2+^-independent spontaneous release more strongly than Ca^2+^-dependent spontaneous release.

### Inhibition of MLCK, but not other CaM targets, closely mimics the distinct effects of CaM inhibition on evoked and spontaneous release

**Figure 6.**
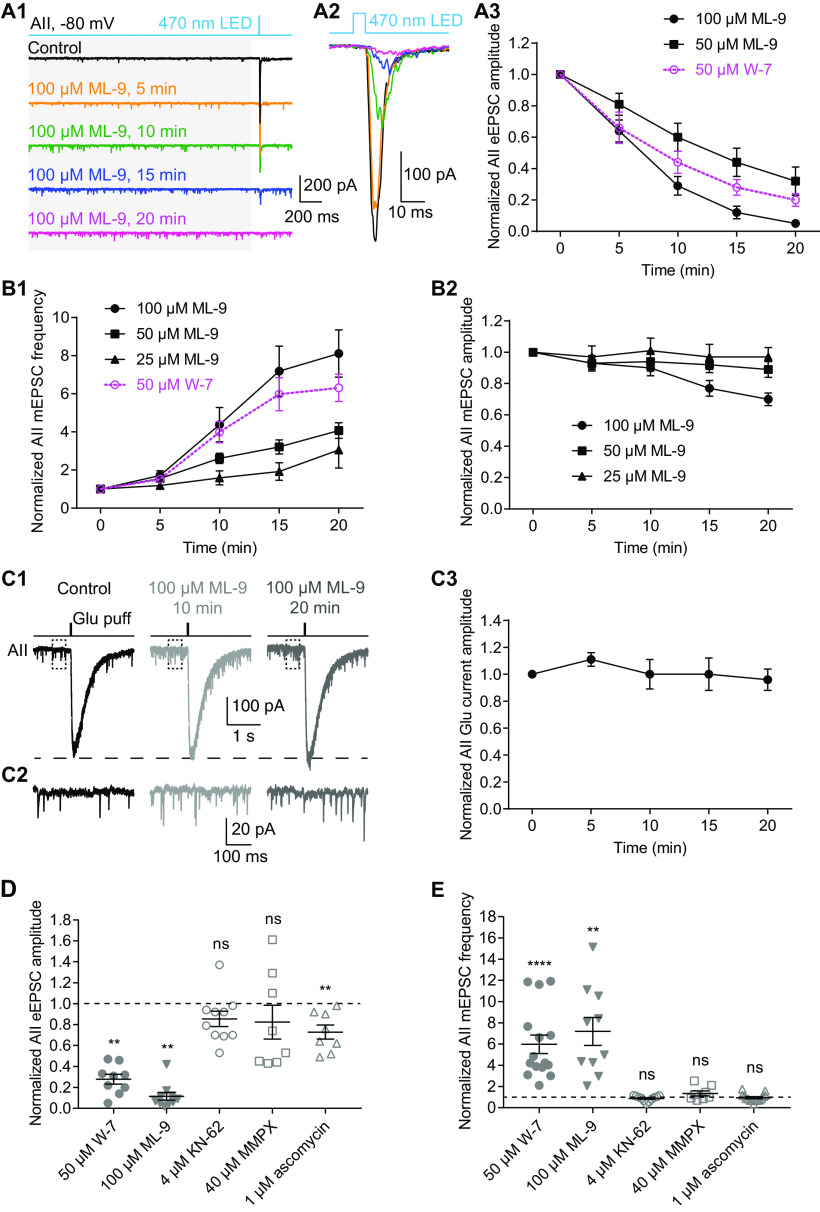
Inhibition of MLCK, but not other CaM targets, differentially regulates evoked and spontaneous release from RBs. ***A1***, Five-millisecond flashes of 470-nm LED were presented to stimulate ChR2-expressing RBs in Pcp2-cre::Ai32 mice. The mEPSCs (in gray background area) and eEPSCs in AIIs were recorded. V_hold_ = −80 mV. Individual traces showed that a specific MLCK inhibitor, ML-9 (100 μm) strongly increased mEPSC frequency and reduced eEPSC amplitude. ***A2***, Average traces of eEPSCs recorded in the same AII in ***A1***. ***A3***, Statistics of the effects of 50 μm (*n* = 5) and 100 μm (*n* = 10) ML-9 on eEPSC amplitude. The amplitudes were normalized to the amplitude at time 0 in each cell before averaging across cells. The data of 50 μm W-7 (adapted from Fig. 2*B3*, superimposed in magenta) were also included for direct comparison. ***B1***, Statistics of the effects of 25 μm (*n* = 7), 50 μm (*n* = 13), and 100 μm (*n* = 10) ML-9 on mEPSC frequency. The frequencies were normalized to the frequency at time 0 in each cell before averaging across cells. The data of 50 μm W-7 (adapted from Fig. 2*C1*, superimposed in magenta) were also included for direct comparison. ***B2***, Statistics of the effects of 25 μm (*n* = 7), 50 μm (*n* = 13), and 100 μm (*n* = 10) ML-9 on mEPSC amplitude. The amplitudes were normalized to the amplitude at time 0 in each cell before averaging across cells. ***C1***, Individual traces showing that ML-9 (100 μm) had no inhibitory effect on AMPA receptor-mediated currents recorded in an AII evoked by glutamate (1 mm) applied onto the AII dendrites at the border of the IPL and GCL. V_hold_ = −80 mV. ***C2***, Magnification of the traces in the dashed line frames of ***C1***, showing increase of mEPSC frequency by ML-9. ***C3***, Statistics of the effects of 100 μm ML-9 (*n* = 4) on the amplitude of glutamate-evoked currents. The amplitudes were normalized to the amplitude at time 0 in each cell before averaging across cells. ***D***, Summary data showing the effects of bath application of W-7 (50 μm; full circles; adapted from Fig. 2*E*), ML-9 (100 μm; full down triangles), KN-62 (4 μm; empty circles), MMPX (40 μm; empty squares), and ascomycin (1 μm; empty up triangles), respectively, for 15 min on the amplitude of eEPSCs recorded in AIIs. In each group of data, the amplitudes were normalized to the amplitude before application of a drug in each cell before averaging across cells. The data were also illustrated as mean ± SEM. Wilcoxon signed-rank tests were used (control vs W-7, *n* = 9, *p* = 0.0039; control vs ML-9, *n* = 10, *p* = 0.0020; control vs KN-62, *n* = 10, *p* = 0.0586; control vs MMPX, *n* = 8, *p* = 0.4609; control vs ascomycin, *n* = 8, *p* = 0.0078); **p < 0.01, ****p < 0.0001; ns: not statistically different. ***E***, Summary data showing the effects of bath application of W-7 (50 μm), ML-9 (100 μm), KN-62 (4 μm), MMPX (40 μm), and ascomycin (1 μm) for 15 min on mEPSC frequency. In each group of data, the frequencies were normalized to the frequency before application of a drug in each cell before averaging across cells. The data were also illustrated as mean ± SEM. Wilcoxon signed-rank tests were used (control vs W-7, *n* = 15, *p* < 0.0001; control vs ML-9, *n* = 10, *p* = 0.0020; control vs KN-62, *n* = 11, *p* = 0.2139; control vs MMPX, *n* = 8, *p* = 0.3828; control vs ascomycin, *n* = 15, *p* = 0.3591); **p < 0.01, ****p < 0.0001; ns: not statistically different.

Given that CaM modulates Ca channels directly (Ben-Johny and Yue, 2014), we wished to determine whether the effect of W-7 was mediated by CaM acting directly on target proteins or indirectly, via a downstream second messenger. Therefore, we recorded simultaneously mEPSCs and ChR2-driven eEPSCs in AII amacrine cells. Strikingly, bath application of a specific MLCK inhibitor, ML-9 (100 μm) had strong, distinct effects on mEPSCs and eEPSCs, reminiscent of the effects of W-7: ML-9 strongly increased mEPSC frequency (Fig. 6*A1*) while dramatically suppressing eEPSC amplitude (Fig. 6*A1*,*A2*). The effects of ML-9 on eEPSCs (*n* = 10 for 100 μm; *n* = 5 for 50 μm; Fig. 6*A3*) and mEPSCs (*n* = 10 for 100 μm; *n* = 13 for 50 μm; *n* = 7 for 25 μm; Fig. 6*B1*) were both time and concentration dependent. ML-9, also, seemed to have a weakly inhibitory effect on mEPSC amplitude, especially at high concentrations (*n* = 10 for 100 μm; *n* = 13 for 50 μm; *n* = 7 for 25 μm; Fig. 6*B2*). We conclude that inhibition of MLCK strongly enhanced spontaneous release from RB axon terminals.

To exclude the possibility that the suppression of eEPSCs by ML-9 was because of a postsynaptic effect, we recorded AMPA receptor-mediated currents evoked by pressure ejection of glutamate (1 mm) onto AII dendrites when CoCl_2_ (1 mm) was included in the external solution to block synaptic transmission. As was the case with W-7, 100 μm ML-9 had little effect on the glutamate-evoked currents (*n* = 4; Fig. 6*C1*,*C3*). Notably ML-9 still increased mEPSC frequency even when Ca channels were blocked with CoCl_2_ in the external solution (Fig. 6*C2*).

We also examined the effects of inhibitors of other downstream CaM targets such as the CaMKII inhibitor KN-62 (4 μm), the PDE1 inhibitor MMPX (40 μm), and the calcineurin inhibitor ascomycin (1 μm). None of these showed significant effects on eEPSCs (Fig. 6*D*) or mEPSCs (Fig. 6*E*) except ascomycin, which slightly reduced the amplitudes of eEPSCs (Fig. 6*D*; Table 2). Ascomycin, however, did not affect mEPSC frequency (Fig. 6*E*), suggesting that it does not act in the pathway inhibited by W-7 and ML-9. Thus, we conclude that inhibition of MLCK, but not other CaM targets, results from inhibiting CaM in RB terminals.

**Table 2:** The effects of inhibitors on AII eEPSC amplitude and mEPSC frequency

	Data	Data structure	Type of test	Power	Mean ± SEM	Number of cells
	Effect on AII eEPSC amplitude					
	Control	Non-normal distribution			1.00 ± 0.00	
	50 μm W-7	Normal distribution			0.28 ± 0.05	9
	100 μm ML-9	Non-normal distribution			0.12 ± 0.04	10
	4 μm KN-62	Normal distribution			0.85 ± 0.07	10
	40 μm MMPX	Non-normal distribution			0.82 ± 0.16	8
	1 μm ascomycin	Normal distribution			0.73 ± 0.07	8
a	Control vs W-7		Wilcoxon signed-rank test	*p* = 0.0039		9
b	Control vs ML-9		Wilcoxon signed-rank test	*p* = 0.0020		10
c	Control vs KN-62		Wilcoxon signed-rank test	*p* = 0.0586		10
d	Control vs MMPX		Wilcoxon signed-rank test	*p* = 0.4609		8
e	Control vs ascomycin		Wilcoxon signed-rank test	*p* = 0.0078		8
	Effect on AII mEPSC frequency					
	Control	Non-normal distribution			1.00 ± 0.00	
	50 μm W-7	Non-normal distribution			5.95 ± 0.86	15
	100 μm ML-9	Normal distribution			7.19 ± 1.32	10
	4 μm KN-62	Normal distribution			0.88 ± 0.07	11
	40 μm MMPX	Normal distribution			1.35 ± 0.23	8
	1 μm ascomycin	Normal distribution			0.94 ± 0.10	15
f	Control vs W-7		Wilcoxon signed-rank test	*p* < 0.0001		15
g	Control vs ML-9		Wilcoxon signed-rank test	*p* = 0.0020		10
h	Control vs KN-62		Wilcoxon signed-rank test	*p* = 0.2139		11
i	Control vs MMPX		Wilcoxon signed-rank test	*p* = 0.3828		8
j	Control vs ascomycin		Wilcoxon signed-rank test	*p* = 0.3591		15

### Inhibition of MLCK also inhibits presynaptic Ca currents and potentiates Ca^2+^-independent spontaneous release

**Figure 7.**
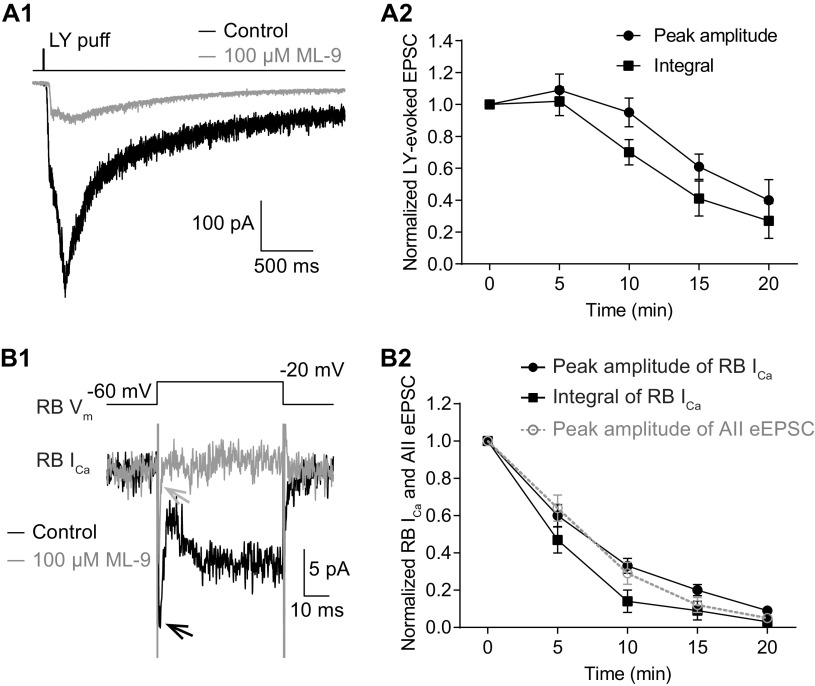
Inhibition of MLCK strongly reduces evoked release by suppressing calcium currents in RBs. ***A1***, Average traces showing that EPSCs recorded in an AII, evoked by puffing LY 341495 (LY), an mGluR6 antagonist, onto the dendrites of RBs located at the OPL, were strongly reduced by application of 100 μm ML-9. V_hold_ = −80 mV. ***A2***, LY-evoked EPSCs decreased over time with bath application of 100 μm ML-9 (*n* = 5). The peak amplitudes and integrals of EPSCs were normalized to the amplitude and integral at time 0, respectively, in each cell before averaging across cells. All the data were illustrated as mean ± SEM. ***B1***, Average traces showing that ML-9 (100 μm) strongly suppressed the voltage step-generated calcium currents (I_Ca_) in an RB. ***B2***, Statistics of the effects of 100 μm ML-9 (*n* = 10) on the peak amplitude and integral of RB I_Ca_. The suppression of I_Ca_ recorded in RBs was closely related to the inhibition of eEPSCs recorded in AIIs (adapted from Fig. 6*A3*, superimposed in gray). All the data were illustrated as mean ± SEM.

**Figure 8.**
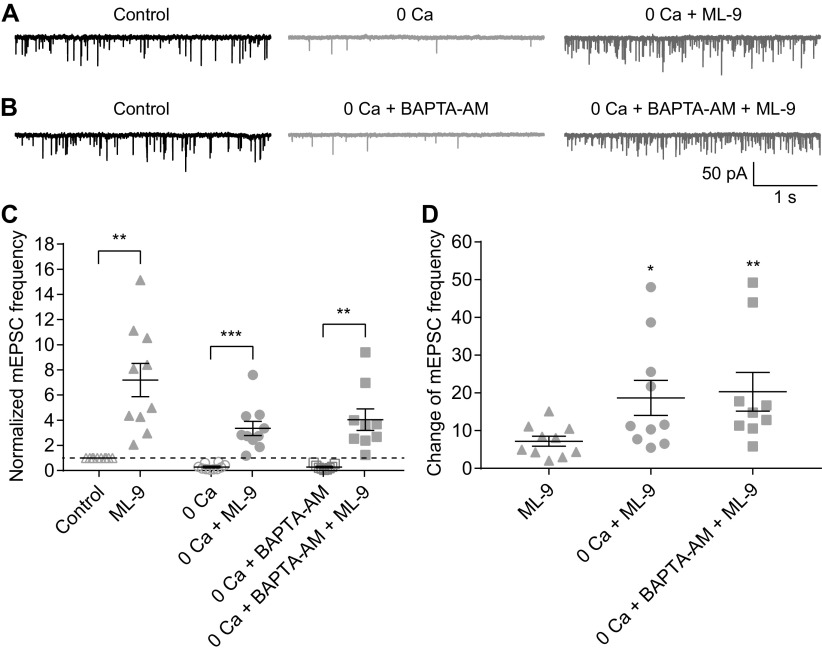
Both Ca^2+^-dependent and Ca^2+^-independent spontaneous release are enhanced, but to different extents, when MLCK is inhibited. ***A***, ***B***, Representative traces showing AII mEPSCs under control, and different combinations of removal of extracellular calcium (0 Ca), 10 μm BAPTA-AM and 100 μm ML-9 conditions. ***C***, Summary data for AII mEPSC frequency under two experimental conditions in ***A*** (empty and full circles) and ***B*** (empty and full squares). The data under control and ML-9 conditions (adapted from Fig. 6*B1*, empty and full triangles, respectively) were also presented for direct comparison. The frequencies were normalized to the frequency under control condition in each cell before averaging across cells. The data were also illustrated as mean ± SEM. Wilcoxon signed-rank tests were used (control vs ML-9, *n* = 10, *p* = 0.0020; 0 Ca + BAPTA-AM vs 0 Ca + BAPTA-AM + ML-9, *n* = 9, *p* = 0.0039) except for comparison of 0 Ca and 0 Ca + ML-9 data by paired Student’s *t* test (*n* = 10, *p* = 0.0001); **p < 0.01, ***p < 0.001. ***D***, Summary data for changes of AII mEPSC frequency after bath application of 100 μm ML-9 for 15 min under control and two experimental conditions. The change in each cell was calculated as the ratio of mEPSC frequencies before and 15 min after application of ML-9. The data were also illustrated as mean ± SEM. Mann–Whitney tests were used (ML-9 vs 0 Ca + ML-9, *p* = 0.0232; ML-9 vs 0 Ca + BAPTA-AM + ML-9, *p* =0.0030); *p < 0.05, **p < 0.01.

We repeated several experiments performed with W-7 using ML-9 to confirm that the two agents exerted broadly similar effects. ML-9 inhibited LY-evoked EPSCs recorded in AIIs (Fig. 7*A1*,*A2*) and Ca currents recorded in RBs (Fig. 7*B1*,*B2*). As well, ML-9 acted as W-7 did under the two experimental conditions in which extracellular and intracellular [Ca^2+^] were altered (compare Figs. 5 and 8; Table 3).

**Table 3:** The effects of ML-9 on AII mEPSC frequency under different conditions

	Data	Data structure	Type of test	Power	Mean ± SEM	Number of cells
	Control	Non-normal distribution			1.00 ± 0.00	10
	100 μm ML-9	Normal distribution			7.19 ± 1.32	10
a	Control vs ML-9		Wilcoxon signed-rank test	*p* = 0.0020		
	0 Ca^2+^ (experimental condition 1)	Normal distribution			0.28 ± 0.07	10
	0 Ca^2+^ + ML-9	Normal distribution			3.35 ± 0.57	10
b	0 Ca^2+^ vs 0 Ca^2+^ + ML-9		paired *t* test	*p* = 0.0001		
	0 Ca^2+^ + BAPTA-AM (experimental condition 2)	Normal distribution			0.28 ± 0.07	9
	0 Ca^2+^ + BAPTA-AM + ML-9	Non-normal distribution			4.04 ± 0.85	9
c	0 Ca^2+^ + BAPTA-AM vs 0 Ca^2+^ + BAPTA-AM + ML-9		Wilcoxon signed-rank test	*p* = 0.0039		
	Relative effect of ML-9 under different conditions					
	ML-9 effect under control condition	Normal distribution			7.19 ± 1.32	10
	ML-9 effect under experimental condition 1	Non-normal distribution			18.69 ± 4.64	10
	ML-9 effect under experimental condition 2	Non-normal distribution			20.31 ± 5.12	9
d	ML-9 (experimental condition 1) vs ML-9 (control condition)		Mann–Whitney test	*p* = 0.0232		
e	ML-9 (experimental condition 2) vs ML-9 (control condition)		Mann–Whitney test	*p* = 0.0030		

0 Ca^2+^: remove extracellular Ca^2+^.

### Inhibition of CaM occludes the potentiating effect of MLCK inhibition on spontaneous release

To determine whether W-7 and ML-9 exerted their effects via the same intracellular signaling pathway (i.e., the CaM-MLCK pathway), we performed an occlusion experiment: following inhibition of CaM with W-7 (>20-min preincubation), we antagonized MLCK with ML-9. If inhibition of CaM reduces MLCK activity, then we expected the effect of ML-9 to be reduced in the presence of W-7. Indeed, the experimental result is in line with this expectation.

**Figure 9.**
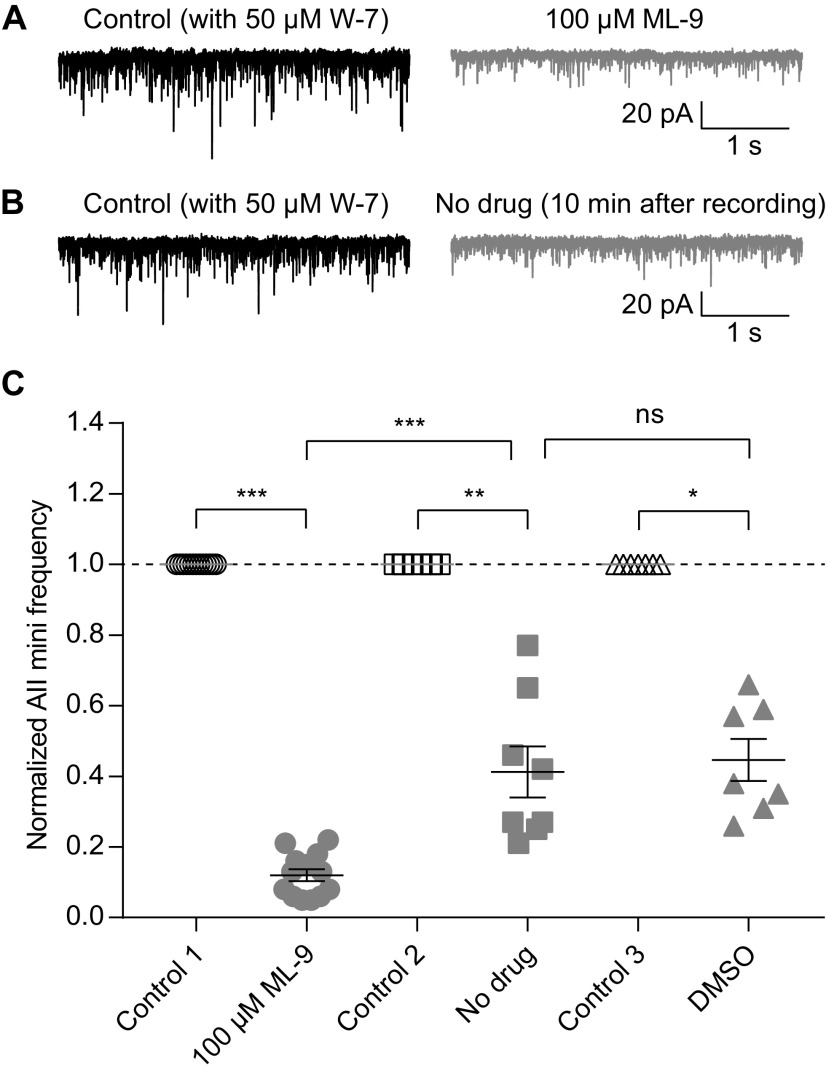
Inhibition of CaM occludes the effect of MLCK inhibition on spontaneous release. ***A***, Representative traces showing that, after preincubation with 50 μm W-7, 100 μm ML-9 did not increase, but instead reduced, AII mEPSC frequency. ***B***, Representative traces showing that, after preincubation with 50 μm W-7, AII mEPSC frequency decreased significantly after 10-min recording. ***C***, Summary data for AII mEPSC frequency under three experimental conditions in ***A*** (empty and full circles), ***B*** (empty and full squares), and DMSO control (empty and full triangles). The frequencies were normalized to the frequency under control condition in each cell before averaging across cells. The data were also illustrated as mean ± SEM. Wilcoxon signed-rank tests (control 1 vs ML-9, *n* = 13, *p* = 0.0002; control 2 vs no drug, *n* = 8, *p* = 0.0078; control 3 vs DMSO, *n* = 7, *p* = 0.0156) or unpaired Student’s *t* test (ML-9 vs no drug, *p* = 0.0001; no drug vs DMSO, *p* = 0.7337) were used for comparison; *p < 0.05, **p < 0.01, ***p < 0.001; ns: not statistically significant.

Specifically, in the presence of 50 μm W-7 (>20-min preincubation), application of 100 μm ML-9 did not increase, but instead reduced, AII mEPSC frequency (control 1 vs ML-9, 1.00 vs 0.12, *n* = 13, *p* = 0.0002; Fig. 9*A*,*C*). As a control for the prolonged recording period, we measured mEPSC frequencies over time in the absence of any drug application and noted that mEPSC frequency declined over time (control 2 vs no drug, 1.00 vs 0.41, *n* = 8, *p* = 0.0078; Fig. 9*B*,*C*), although the decline was less pronounced than in the W-7 + ML-9 condition (ML-9 vs no drug, *p* = 0.0001; Fig. 9*C*). DMSO alone did not have any additional effect on mEPSC frequency compared with the control condition (control 3 vs DMSO, 1.00 vs 0.45, *n* = 7, *p* = 0.0156; DMSO vs no drug, *p* = 0.7337; Fig. 9*C*). We conclude, then, that the CaM-MLCK pathway is involved in regulating spontaneous release from RBs.

## Discussion

We monitored the dynamics of transmission at RB→AII synapses to examine the effects of modulating presynaptic CaM and its target proteins and made two major findings. One, we found that inhibition of CaM strongly reduced evoked release by suppressing presynaptic Ca currents while simultaneously potentiating both Ca^2+^-dependent and Ca^2+^-independent spontaneous release (Figs. 2-5). Two, the effect of inhibiting CaM seemed to be mediated by inhibition of MLCK but not other CaM downstream targets (including CaMKII, PDE1 and calcineurin; Figs. 6-9). Thus, CaM, via activation of MLCK, suppresses spontaneous release and promotes evoked release at retinal ribbon synapses.

### Pharmacological agents

Concerns about the specificity of pharmacological agents are common and vex experimentalists. We made three observations that should assuage such concerns. One, our immunohistochemical analysis revealed extremely high expression of CaM in RB axon terminals. Even so, application of CaM antagonists such as W-7 might exert off-target effects. Therefore, we tested a structurally dissimilar CaM antagonist, CMZ (Gietzen et al., 1982), in our preparation and found that at concentrations of 50–100 μm, it too reduced evoked release, although to a lesser extent (Fig. 2*E*). We also tested the effects of CALP1, a CaM agonist (Villain et al., 2000). CALP1 reduced spontaneous release, especially after removal of extracellular Ca^2+^, whereas it had no effect on evoked release (Fig. 2*E*,*F*), which might be explained by the almost full activation of CaM by Ca^2+^ sufficient to saturate endogenous buffers (Mehta et al., 2014) under our experimental conditions. Three, inhibition of MLCK also bidirectionally regulated evoked and spontaneous release and the effect could be occluded by preinhibition of CaM, which would in turn support the conclusion that neurotransmitter release at RB→AII synapse is modulated by CaM.

Although CaBP5 is expressed in mouse retinal RBs and is suggested to be involved in neurotransmitter release since it interacts with Munc18-1 and myosin VI (Haeseleer et al., 2000; Rieke et al., 2008; Sokal and Haeseleer, 2011), to our knowledge, there is no evidence showing that W-7 could antagonize CaBP5. It has been shown that CaBP5 has only a relatively weak effect on inactivation of Ca currents when cotransfected with calcium channels in HEK293T cells (Rieke et al., 2008). In contrast, we found that W-7 inhibited RB I_Ca_ strongly. This would indicate that, even if W-7 might inhibit CaBP5, it exerted its inhibitory effect on RB I_Ca_ by primarily targeting CaM, rather than CaBP5.

### CaM and its downstream targets at ribbon synapses

It has been proposed that CaMKII may be the downstream target of CaM in regulation of evoked release (Llinás et al., 1985; Pang et al., 2010). CaMKII has been found located close to synaptic ribbons (Uthaiah and Hudspeth, 2010; Kantardzhieva et al., 2012), and it has been shown to phosphorylate syntaxin 3B, the retinal isoform of syntaxin and an essential component of the core SNARE complex mediating vesicle fusion (Liu et al., 2014). We were surprised, then, that the CaMKII inhibitor KN-62 only had a small (not significant) effect on either evoked or spontaneous release at RB→AII synapses. In contrast, the MLCK inhibitor ML-9 exerted strong effects on both two forms of release.

MLCK has been suggested to be involved in regulation of neurotransmitter release, with opposing effects observed at different synapses (Mochida et al., 1994; Mochida, 1995; Ryan, 1999; Polo-Parada et al., 2005; Srinivasan et al., 2008), although there is a study arguing that MLCK is not a regulator for synaptic vesicle trafficking in hippocampal neurons (Tokuoka and Goda, 2006). In addition, MLCK accelerates vesicle endocytosis at the calyx of Held and hippocampal synapses (Yue and Xu, 2014; Li et al., 2016) and enhances ribbon replenishment in cone photoreceptors (Van Hook et al., 2014). Our findings support the role that MLCK plays in regulating neurotransmitter release. We could imagine that inhibition of MLCK slowed down the replenishment of vesicle pools and reduced subsequent evoked release by making the readily-releasable pool (RRP) of vesicles smaller. Potentially, reducing the RRP size could shunt vesicles into a spontaneously-releasing pool.

### CaM regulates evoked and spontaneous release differentially

Recent studies have showed selective molecular regulation of evoked and spontaneous release. Proteins such as RIM1 and RBPs, modulate evoked, but not spontaneous, release (Calakos et al., 2004; Acuna et al., 2015; Robinson et al., 2019), whereas proteins such as Vti1a, VAMP7, Doc2, and copine-6, modulate spontaneous, but not evoked, release (Groffen et al., 2010; Pang et al., 2011; Ramirez et al., 2012; Bal et al., 2013; Liu et al., 2018). Despite this, it is clear that evoked and spontaneous release do not use completely nonoverlapping molecular machinery: both are mediated by the canonical SNARE complex including syntaxin and SNAP-25 (Südhof, 2012). Additionally, synaptotagmin-1 and complexins have been shown to control both evoked and spontaneous release (Maximov and Südhof, 2005; Huntwork and Littleton, 2007; Xu et al., 2009; Yang et al., 2013; Bai et al., 2016; Grassmeyer et al., 2019; López-Murcia et al., 2019). Indeed, knock-out of complexin three abolishes fast, synchronous release while enhancing spontaneous release at RB→AII synapses (Mortensen et al., 2016). Our present study thus likely would add CaM as well as MLCK into the family of dual regulators of evoked and spontaneous release.

Inhibition of CaM by W-7 reduced evoked release by suppressing Ca^2+^ influx into RB axon terminals (Fig. 4). CaM can directly bind several types of VGCCs and thereby regulate their activity (Ben-Johny and Yue, 2014). Generally, CaM is thought to mediate Ca^2+^-dependent inactivation (CDI) of Ca channels (Ben-Johny and Yue, 2014; Pangrsic et al., 2018). Ca currents in RB terminals, however, show little CDI (Singer and Diamond, 2003; Jarsky et al., 2011; Dolphin and Lee, 2020), and here W-7 (i.e., inhibiting CaM) inhibited rather than potentiated Ca currents. Thus, CaM likely does not bind to Ca channels in RB terminals directly.

It has been reported that W-7 can inhibit VGCCs in some non-neuronal systems such as ciliary membrane of *Paramecium* and smooth muscle from rat vas deferens (Hennessey and Kung, 1984; Nakazawa et al., 1993). CMZ inhibits VGCCs in different smooth muscle cells (Klöckner and Isenberg, 1987; Nakazawa et al., 1993; Sunagawa et al., 1999), but it has no effect on Ca currents in *Paramecium* (Ehrlich et al., 1988). The inhibitory effects of W-7 and CMZ on VGCCs are suggested to be CaM-independent and likely because of direct actions of these drugs on VGCCs, based on the limited evidence that exogenous CaM has no effect on VGCCs and that CaMKII antagonists, when applied either extracellularly or intracellularly, do not block the effect of CMZ on VGCCs (Klöckner and Isenberg, 1987; Ehrlich et al., 1988; Sunagawa et al., 1999).

Similar results have also been observed in our study: activation of CaM by CALP1 did not enhance evoked release, and neither CaMKII nor PDE1 seemed to be involved in regulating neurotransmitter release from RBs. Note, however, that W-7 and CMZ likely have distinct effects on different CaM-dependent pathways. For example, CMZ, at the concentration of 1 μm, dramatically inhibits the activity of CaM-dependent PDE, while W-7, even at the concentration as high as 100 μm, only has a very small effect (Ehrlich et al., 1988). By contrast, it may be possible that W-7 has a stronger effect on other downstream targets of CaM, such as MLCK, than CMZ. Indeed, we found that W-7 inhibited evoked release from RBs more strongly than CMZ, and MLCK was likely the mediator of the effects observed. It has been shown that ML-9 (and also its structural analog, ML-7) inhibits VGCCs in hippocampal neurons, and this effect may be independent of MLCK since it is not mimicked by wortmannin, a relatively non-specific MLCK inhibitor (Tokuoka and Goda, 2006). We could not exclude the possibility that both W-7 and ML-9 inhibit VGCCs directly. But it is unlikely to be true since ML-9 not only closely mimicked the effects of W-7 on VGCCs and evoked release but also on Ca^2+^-independent spontaneous release (Figs. 2, 5, 6, 8), which is not related to VGCCs. Further, preincubation of W-7 completely occluded the potentiating effect of ML-9 on spontaneous release (Fig. 9), indicating that these two drugs exerted their effects via the same (CaM-MLCK) pathway.

Evidence for direct interactions between MLCK and Ca channels in RB terminals is not apparent in the literature, and therefore it will be interesting to explore how MLCK controls the activity of Ca channels and Ca^2+^-dependent exocytosis in the future. But generally, our present observations support the notion that CaM promotes evoked release, which is consistent with other studies (Chamberlain et al., 1995; Chen et al., 1999; Junge et al., 2004; Pang et al., 2010).

Unique mechanisms of spontaneous neurotransmitter release have received significant attention recently (Kavalali, 2015). Spontaneous release is largely dependent on Ca^2+^ influx through VGCCs or Ca^2+^ efflux from internal stores (Kaeser and Regehr, 2014; Schneggenburger and Rosenmund, 2015; Williams and Smith, 2017). Indeed, we observed that spontaneous release was reduced by ∼70% (Figs. 5, 8) when the extracellular Ca^2+^ was removed and the intracellular Ca^2+^ was buffered by the membrane permeable Ca^2+^ chelator BAPTA-AM. Our observations, however, indicate that there is a Ca^2+^-independent component of spontaneous release that is poorly understood. Given that spontaneous energy fluctuations that overcome the energy barrier for vesicle fusion can trigger spontaneous exocytosis (Schneggenburger and Rosenmund, 2015), we believe it possible that inhibition of CaM or MLCK somehow reduces the energy barrier to exocytosis, much as Ca^2+^ binding to synaptotagmin does.

## Synthesis

Reviewing Editor: Juan Burrone, King’s College London Decisions are customarily a result of the Reviewing Editor and the peer reviewers coming together and discussing their recommendations until a consensus is reached. When revisions are invited, a fact-based synthesis statement explaining their decision and outlining what is needed to prepare a revision will be listed below. The following reviewer(s) agreed to reveal their identity: Amy Lee.

The authors show that calmodulin, acting through MLCK, has opposite effects on evoked and spontaneous neurotransmitter release at the ribbon synapse of retinal bipolar neurons. Overall, the experiments are elegant and well designed. In particular, the effects on synaptic transmission are novel and of interest to the field. However, one concern in this study is the specificity of the drugs used and, therefore, of the conclusions reached regarding the molecular mechanisms. Below, are the comments and concerns of the reviewers that the authors should address.

Reviewer 1

This manuscript addresses the effects of pharmacological inhibitors on synaptic transmission from the rod-bipolar cell to the Aii amacrine cell. The authors find that the CM inhibitor W7 and the MLCK inhibitor, ML-9 increased mEPSC frequency, while decreasing Ca-current and evoked EPSCs. The authors conclude that CaM acts via MLCK to modulate glutamate release from RBCs. Overall, the work is carefully done and the results novel, but I am concerned about the specificity of some of the reagents. In particular, RBCs are known to have an abundance of the Calmodulin-like protein CaBP5, which may cross-react with both antibodies and inhibitors of calmodulin. Unless the authors have a strong rationale for ruling out CaBP5, this possibility needs to be considered and discussed as an alternative explanation.

Specific comments:

1) The antibody that the authors use for calmodulin IHC is raised against the C-terminus to CaM, which is similar to CaBP5. The authors should verify the antibody doesn’t react with CaBP5 or identify an antibody that can distinguish between the two.

2) The authors mention results with calmidazolium, which were not shown, but had apparently a more modest effect on mEPSC frequency. These should be shown.

3) Many of the inhibitors are poorly soluble in water. Were these dissolved in DMSO or other carrier solvent before dissolving in water? If so, then were DMSO controls run?

4) In figure 2B3, it would be good to see a 0 W7 trace to know what the normal level of rundown is for these experiments.

5) The Ca-current rundown under control conditions under voltage clamp should be shown as well.The effect on Ca-current is surprisingly large, with almost complete abolition of calcium current. Is it possible that some of this is rundown?

6) CALP1 had only modest effects on mEPSC frequency, which was attributed to near full activation of CaM at rest. If true, then the effect on Ca-independent mEPSCs would be expected to be more dramatic. Have the authors tested this?

Reviewer 2

In this study, the authors investigate a potential role for calmodulin (CaM) in regulating synaptic transmission between rod bipolar cells and AII amacrine cells. They find that pharmacological inhibitors of CaM reduce voltage-gated Ca2+ channel (VGCC)-mediated currents and Ca2+-dependent evoked release, while potentiating spontaneous release. Based on these findings, the authors conclude that CaM-dependent activation of myosin light chain kinase (MLCK) promotes supports the activity of VGCCs and evoked release, and at the same time, suppresses spontaneous transmission.

The experiments are expertly executed and presented very clearly. However, there are a number of concerns that should be addressed.

Major concerns:

1) As the authors acknowledge, there are caveats to making conclusions based solely on the pharmacological actions of drugs. W-7 has been shown to inhibit VGCCs (PMID 6747535), and there is some evidence that this could be due to a direct CaM-independent action on VGCCs (PMID 8495236). The results are still pretty interesting in replicating these early findings of effects of W-7, but are not entirely consistent with the authors’ conclusion that W-7 is acting through CaM to suppress VGCC activity. Other results presented in this study further support for a direct action of W-7 on inhibiting VGCCs: (a) CALP1, a CaM agonist, had no effect on the eEPSCs (Fig.2) and (b) calmidazolium had similar but weaker effects on eEPSCs (data not shown), and this drug is also known to inhibit L-type VGCCs (PMID: 10511122). CaM inhibition could secondarily inhibit VGCCs, but as the authors acknowledge, it is not clear how since normally CaM promotes inactivation of VGCCs.

The parallel effects of W-7 and the MLCK inhibitor ML-9 are used to justify the interpretation that CaM acts via MLCK. However, MLCK inhibitors also may directly inhibit neuronal VGCCs (PMID 17093082). W-7 could directly suppress the activity of VGCCs, while MLCK inhibition could influence spontaneous and/or evoked release through other mechanisms. Showing that W-7 occludes the effects of ML-9 (or vice versa) would help mitigate this concern. Alternatively, the authors could test the ability of calmidazolium to occlude the effect of W-7, or wortmannin (another MLCK inhibitor) to occlude effects of ML-9.

2) Fig.1,3: The diffuse localization of CaM and coincidence of CaM and RIBEYE labeling is used as evidence that CaM is positioned to regulate ribbon- and non-ribbon-mediated release. The lack of immunostaining when primary antibodies are excluded (Fig.2A2) is used as the negative control for this assertion. This experiment demonstrates that the secondary antibodies do not label non-specifically, but does not address the specificity of the primary antibodies for their targets. The absence of immunoreactivity upon pre-adsorbing the primary CaM antibody with CaM, but not with a non-relevant protein of similar size, would be a stronger control. However, the authors could simply omit the CaM immunostaining in Fig.1 and Fig.3 without affecting their conclusions. CaM has been found in most presynaptic preparations, so it is not far-fetched that it is abundant also in RB terminals.

Minor comments:

Figure 1: The image of the alexa-filled AII AC needs some adjustment to be able to see the filled cell. Perhaps use a different color other than magenta (like white).

Figure 4: The images in E1 are too dim to see changes in GCaMP fluorescence. Also, there is not a scale bar for these images.
